# Living with hypoparathyroidism: development of the Hypoparathyroidism Patient Experience Scale-Impact (HPES-Impact)

**DOI:** 10.1007/s11136-020-02607-1

**Published:** 2020-08-24

**Authors:** Meryl Brod, Laura Tesler Waldman, Alden Smith, David Karpf

**Affiliations:** 1 The Brod Group, 219 Julia Ave., Mill Valley, CA 94941 USA; 2 Ascendis Pharma, Inc., Palo Alto, CA USA

**Keywords:** Hypoparathyroidism, Patient-reported outcome measures, Quality of life, Adult

## Abstract

**Purpose:**

Hypoparathyroidism (HP) is a rare endocrine disorder characterized by absent or inappropriately low levels of circulating parathyroid hormone (PTH). Research indicates that HP patients on conventional therapy may have a reduced quality of life. The study’s purpose was to develop a new disease-specific measure of the impacts of hypoparathyroidism on functioning and well-being and provide evidence for its content validity based on rigorous qualitative research methodologies for patient-reported outcomes development.

**Methods:**

Semi-structured, individual concept elicitation (CE) interviews were conducted with 5 clinical experts and 42 adults in the USA with HP to identify impacts of relevance and importance to the target population. Transcripts were coded and analyzed using an adapted grounded theory approach common to qualitative research. Following item generation, the draft measure was cognitive debriefed in an independent sample of 16 adults with HP.

**Results:**

Analyses identified four impact domains: physical functioning, including ability to exercise (*n* = 32, 76%) and mobility (*n* = 21, 50%); daily life, including ability to do things around the home (*n* = 33, 79%), and interference with work productivity (*n* = 18, 43%); psychological well-being, including feeling anxious/anxiety (*n* = 34, 81%) and frustrated (*n* = 27, 64%); and social, including ability to participate in social activities (*n* = 33, 79%) and relationships (*n* = 32, 76%). Twenty-seven impacts were identified and included in the preliminary measure. After the cognitive debriefing, a validation-ready, 26-item Hypoparathyroidism Patient Experience Scale-Impact (HPES-Impact) was generated.

**Conclusion:**

Findings provide substantial evidence of content validity for the validation-ready HPES-Impact in adults with HP.

## Background

Hypoparathyroidism (HP) is a rare endocrine disorder characterized by absent or inappropriately low levels of circulating parathyroid hormone (PTH) [1, 2]. Low levels or the absence of PTH circulating in the bloodstream can lead to hypocalcemia (low blood calcium levels), hyperphosphatemia (elevated blood phosphate levels), hypercalciuria (elevated urinary calcium levels), and overly-mineralized bone [1, 3]. Significant physical and cognitive symptoms are associated with the condition, including fatigue, muscle cramping/spasms, paresthesia (sensation of tingling and/or numbness), cognitive dysfunction, and sleep disturbances [1, 4–8].

Based on data from 2007–2009, the prevalence of HP in the USA has been estimated to be between 70,000—115,000 patients [1, 9–11]. The most common sources of HP are surgery in the neck region [2, 11], autoimmune disease [2, 12], and idiopathic [2]. HP is typically treated with oral calcium and vitamin D supplements [1–3, 13]. PTH [1–84] replacement therapy has also been approved by the US Food & Drug Administration (FDA) for adults who do not respond to conventional therapy [1, 14].

Research indicates that patients with HP on conventional and/or PTH replacement therapy may have a reduced quality of life (QOL) [1, 5, 15–17]. In multiple studies, patients on conventional therapy have had lower SF-36 [5, 6, 18, 19] and WHO-5 Well-Being Index [6] scores compared with the normative reference range and trial controls [6, 18] One study found that patients with post-surgical HP had significantly worse global complaint and subscale scores for anxiety, phobic anxiety, somatization, and their physical equivalents (e.g., heart palpitations) compared with patients who were post-thyroid surgery with intact parathyroid functioning [7]. Another study similarly reported that patients with HP scored worse on the symptom score for anxiety and depression, and the total Hospital Anxiety and Depression score compared with the normative population [18].

Studies of patients on PTH replacement therapy are limited and their findings have varied. In an open-label, uncontrolled PTH replacement study in the USA, significant improvements in the majority of domains of the SF-36 were reported [19]. However, in a randomized, double-blind, placebo-controlled study of PTH replacement therapy in Denmark, no significant difference between placebo and treatment groups was observed in SF-36 and WHO-5 Well-Being Index scores [5]. Although no significant between-group differences were observed in another randomized, double-blind, placebo-controlled study of PTH replacement therapy in the USA, there were significant improvements in multiple domains of the SF-36 for those in the treatment group compared with the placebo group; however, some of these scores remained low in comparison with the normal population [20].

As measured by the SF-36, QOL has further been reported to be reduced for patients with post-surgical HP compared to patients with other sources of HP [18, 20]. Since most patients with post-surgical HP also have post-surgical hypothyroidism, limited data is available on the extent to which the reduced QOL is due to HP or the combination of HP and hypothyroidism [6].

HP may also negatively impact daily life and work productivity. In a web-based survey of 374 adults with HP in the USA, 45% reported significant interference with their life due to HP [8]. Approximately one-fifth reported that symptoms associated with HP directly influenced a change in work status. Respondents further reported an increase in being unemployed, on disability, or retired, and a decrease in working either full- or part-time. Similarly, in a study of patients with HP in Norway, 40% reported receiving either permanent or temporary social security benefits compared with only 14% of the country’s general adult population [18].

Although previous research has provided evidence that QOL may be reduced in patients with HP, the validated questionnaires used in these studies were not disease-specific, and did not assess a number of documented symptoms associated with this condition, such as cognitive deficits, fatigue, or decreased muscle strength [15, 21]. In addition, owing to the lack of disease-specific questionnaires in previous research, impacts associated with these symptoms may also have been inadequately assessed.

The purpose of this study was to develop disease-specific measures of the symptoms and health-related QOL (HRQOL) impacts of HP, and provide evidence for the content validity of the measure based on rigorous qualitative research methodologies for patient-reported outcomes (PRO) development [22–27]. The development of the first of these measures, the Hypoparathyroidism Patient Experience Scale-Symptom (HPES-Symptom) has been previously reported [28]. This paper describes the development of the Hypoparathyroidism Patient Experience Scale-Impact (HPES-Impact). This paper also describes the development of a preliminary theoretical model to identify relationships among key concepts, impacts, and modifiers related to the new PRO impact measure. Condition-specific instruments reportedly have greater face validity, are more responsive to change over time, and can be more useful to both clinicians and researchers to assess the impact of treatment on patients with HP [22–25].

## Methods

The HPES-Impact was developed in accordance with the FDA guidance [29] on best research practices for PRO measure development [22–25]. The methods used to carry out this research, including eligibility criteria, have been described in detail in the HPES-Symptom article [28]. To briefly summarize, qualitative, individual concept elicitation (CE) interviews conducted by telephone with clinical experts and adults with HP were used to identify key concepts, the impacts of HP, that were both relevant and important to people with this condition to inform the development of the PRO measure and develop the preliminary theoretical model [24]. The clinical experts were well-established endocrinologists in the USA and Denmark with publications on HP who were currently treating patients with HP and had been recruited through a key opinion leader. Patient participants were adults in the USA with a diagnosis of HP who had been stable on standard-of-care (oral calcium and vitamin D supplements) and/or short-acting PTH replacement therapy for at least 3 months.

They were recruited via a national hypoparathyroidism organization and required to verify their eligibility by answering telephone screening questions and providing documentation of their diagnosis.

CE interviews with clinical experts and patient participants were completed by trained qualitative research interviewers familiar with HP who used a semi-structured interview guide, based on a literature review and discussions with clinical experts, that explored the signs and symptoms of HP and their impacts on QOL. Interviews lasted approximately one hour. Data were qualitatively analyzed through an adapted grounded theory approach, entailing developing and refining a theory based on concepts derived during the research process [26]. To carry out this analysis, participant and clinical expert transcripts were analyzed for content using Dedoose (www.dedoose.com), a qualitative analysis software program. First, the research team developed a preliminary code list based on the discussion guides’ sensitizing (initial, general) concepts [30] and subsequently revised the list based on concepts derived from the interviews during the coding process. For the portion of the study related to the development of the HPES-Impact measure, the overarching theme was the impacts associated with HP, and the key concepts within these were the specific impacts reported by participants and/or clinical experts. Within each of these study populations, each transcript was initially read, then coded, and reviewed multiple times to ensure accuracy and consistency. Transcripts were coded in the order in which interviews had been conducted, and emerging concepts were incorporated into the coding scheme as they were identified. Prior transcripts were then re-examined for the presence of newly emerged concepts. The coding scheme was refined throughout the analysis, including merging, modifying, or deleting codes as the researcher team gained further insights into particular concepts and the extent to which study participants considered them to be relevant and important. Lastly, following this iterative process and a series of internal deliberations to reach consensus among the research team members, the coded concepts were organized into larger categories of major themes (the impact domains).

Thematic saturation, defined as that point in time when no new major themes or subthemes emerged from the interviews and sufficient data had been collected to comprehensively identify, develop and understand the depth, range and relationships of the concepts and categories of interest [22, 27], was evaluated and confirmed through the development of a saturation grid populated with the key concepts discussed in each patient interview. A total of 101 impacts associated with the disease burden of HP emerged from the participant interviews. After the 14^th^ participant interview, 75% of impacts had been discussed, and by the 24^th^ interview, 95% of impacts were covered. Assessment of additional saturation criteria, including the frequency, severity and bothersomeness described by participants who reported experiencing each impact provided confirmatory evidence that saturation had been reached.

To address the question raised in the literature about the extent to which impaired QOL may be due to HP as a standalone condition versus HP combined with hypothyroidism, comparative frequencies for the overall endorsement rates of impacts were examined based on the proportions of participants with vs. without hypothyroidism. Differences of at least 20% in the proportions of participant endorsement rates between these subgroups were considered to be potentially meaningful. However, meaningful difference was not explored in the interviews; therefore, the 20% threshold cannot be considered a clinically meaningful difference.

A three-day, in-person item generation meeting was held by the project research team (*n* = 4). In accordance with FDA guidelines and good practices for PRO measure development, the team sought to establish criteria to identify impacts that were important and relevant to patients, including patients with varying levels of disease severity and demographic characteristics [22–25, 29], as well as patients with or without hypothyroidism as a comorbidity. All issues that did not fulfill these criteria were categorized as minor, distal, or a modifier.

Based on decisions regarding major and minor impacts, a theoretical model was developed for the relationships among the key concepts of interest, and to identify potential modifiers [22]. Thus informed by the qualitative analysis, the major impacts identified, and the preliminary theoretical model, the team then generated the draft items and created an item definition table, which specified the intent of each item and acceptable synonyms, using the language of the participants as closely as possible.

Following item generation, cognitive debriefing (CD) interviews were conducted to reach consensus on the appropriate item format, structure, and wording, to ensure that items, response options, and instructions were clear and relevant, the recall period was appropriate, and the content was comprehensive [22, 25]. The CD interviews were conducted by telephone in an independent sample of patients with HP who met the same eligibility criteria as the CE interview sample using an item-by-item structured interview guide with probes for clarification. Participants were instructed to complete the measure within 24–48 h of their scheduled interview. This timeframe was chosen since it would be recent enough to avoid recall issues, while also ensuring that participants would have ample time to complete the measure without feeling pressured to finish it immediately prior to the interview. After the CD interviews were completed, a draft version of the measure ready for evaluation of measurement properties was produced, and the preliminary theoretical model was updated to incorporate the interview findings.

The study was approved by an independent Institutional Review Board (IRB), Copernicus Group IRB, located in Cary, North Carolina, USA. Informed consent was obtained from all patient participants.

## Results

### Concept elicitation

#### Sample description

Five expert interviews were conducted with well-established physicians in the USA (*n* = 3, 60%) and Denmark (*n* = 2, 40%). The physicians specialized in endocrinology (*n* = 4, 80%) and endocrinology and internal medicine (*n* = 1, 20%) had practiced in their clinical specialty for an average of 20.1 (range 6–35) years, and had been treating patients with HP for an average of 16.4 (range 5–30) years.

A detailed description of the demographic characteristics of the patient study sample has previously been reported [28]. To briefly summarize these findings, 42 participants were included in data analysis. The majority were female (*n* = 35, 83%) and had post-surgical HP (*n* = 36, *n* = 86%). On average, participants had HP for 14 (range 1–49) years, with an average of 5 (median 4, range 0–20) comorbidities in addition to their HP, and used 5 (range 0–14) prescription medications.

#### Impacts

All but one study participant reported that they continued to experience QOL impacts from their HP despite being on treatment, although many reported a reduction in the severity and/or frequency of these impacts. Participants and clinicians reported 14 HP impacts on physical functioning (Table 1), including impaired ability to exercise (*n* = 32, 76%), impaired mobility (*n* = 21, 50%), and being less physically active than one used to be/wants to be (*n* = 22, 52%).

**Table 1:** Patient- and clinician-reported impacts of hypoparathyroidism on physical functioning

Physical functioning *n* (%)	Comorbidity status		Patient total (*n* = 42)	Clinician total (*n* = 5)
	Has hypothyroidism (*n* = 31)	Does not have hypothyroidism (*n* = 11)		
Inability to exercise	23 (74)	9 (82)	32 (76)	2 (40)
Impaired mobility	14 (45)	7 (64)	21 (50)	0 (0)
Impaired ability to walk	12 (39)	5 (45)	17 (40)	0 (0)
Other forms of impaired mobility	8 (26)	3 (27)	11 (26)	0 (0)
Less physically active than used to be/wants to be	18 (58)	4 (36)	22 (52)	0 (0)
Need a long time to recover after activities	13 (42)	3 (27)	16 (38)	0 (0)
Fine motor skills	8 (26)	1 (9)	9 (21)	0 (0)
Less physical stamina	7 (23)	1 (9)	8 (19)	2 (40)
Inability to lift heavy things	2 (6)	2 (18)	4 (10)	0 (0)
Impaired ability to climb stairs	3 (10)	1 (9)	4 (10)	0 (0)
Impaired ability to sit	3 (10)	1 (9)	4 (10)	0 (0)
Never feels "normal"/always has symptoms	4 (13)	0 (0)	4 (10)	0 (0)
Negative impact on/worsening of comorbidities	2 (6)	0 (0)	2 (5)	0 (0)
Weakened immune system	1 (3)	1 (9)	2 (5)	1 (20)
Balance issues	1 (3)	0 (0)	1 (2)	0 (0)
Doesn't feel like oneself	1 (3)	0 (0)	1 (2)	0 (0)

Participants and clinicians reported a total of 28 HP impacts on daily life (Table 2), including ability to do things around the home (*n* = 33, 79%), needing to take breaks/pace yourself when doing activities (*n* = 26, 62%), and interference with work productivity (*n* = 18, 43%).

**Table 2:** Patient- and clinician-reported impacts of hypoparathyroidism on daily life

Daily life *n* (%)	Comorbidity status		Patient total (*n* = 42)	Clinician total (*n* = 5)
	Has hypothyroidism (*n* = 31)	Does not have hypothyroidism (*n* = 11)		
Impaired ability to do things around the home	25 (81)	8 (73)	33 (79)	2 (40)
Unable to do as much as used to/wants to	22 (71)	6 (55)	28 (67)	0 (0)
Interference with travel	21 (68)	6 (55)	27 (64)	2 (40)
Need to take breaks/pace yourself when doing activities	20 (65)	6 (55)	26 (62)	2 (40)
Interference with work productivity	17 (55)	1 (9)	18 (43)	5 (100)
Need to stop what you're doing due to symptom onset	12 (39)	4 (36)	16 (38)	0 (0)
Impaired ability to complete complex/detail-oriented tasks	12 (39)	3 (27)	15 (36)	2 (40)
Need to plan day around symptoms	10 (32)	5 (45)	15 (36)	0 (0)
Impaired ability to do leisure activities/hobbies	12 (39)	2 (18)	14 (33)	0 (0)
Daily life disrupted by medical visits/hospitalizations	11 (35)	2 (18)	13 (31)	1 (20)
Impaired ability to do errands	10 (32)	3 (27)	13 (31)	0 (0)
Unable to do things for oneself/need help from others	8 (26)	2 (18)	10 (24)	0 (0)
Impaired ability to be outside on warm/hot days	8 (26)	1 (9)	9 (21)	0 (0)
Impaired ability to drive	6 (19)	3 (27)	9 (21)	1 (20)
Impaired ability to go out	8 (26)	0 (0)	8 (19)	0 (0)
Poor overall quality of life	8 (26)	0 (0)	8 (19)	3 (60)
Impaired ability to do personal care	6 (19)	2 (18)	8 (19)	0 (0)
Takes longer to complete tasks	6 (19)	2 (18)	8 (19)	1 (20)
Have to rest all day at times	6 (19)	1 (9)	7 (17)	0 (0)
Interference with school	4 (13)	3 (27)	7 (17)	3 (60)
Impaired ability to get up in the morning	5 (16)	1 (9)	6 (14)	0 (0)
Unable to do tasks as well as used to/wants to	5 (16)	1 (9)	6 (14)	4 (80)
Need to go to bed early	5 (16)	0 (0)	5 (12)	0 (0)
Impaired caregiving ability	2 (6)	1 (9)	3 (7)	1 (20)
Hard to get through the day	3 (10)	0 (0)	3 (7)	0 (0)
Lack of motivation to do things	3 (10)	0 (0)	3 (7)	0 (0)
Accident-prone	2 (6)	0 (0)	2 (5)	0 (0)
Slow to get going in the morning	0 (0)	1 (9)	1 (2)	0 (0)

Participants and clinicians reported a total of 36 psychological HP impacts (Table 3), including feeling anxious/having anxiety (*n* = 34, 81%), frustrated (*n* = 27, 64%), depressed/sad (*n* = 26, 62%), and reduced self-confidence/self-image (*n* = 19, 45%).

**Table 3:** Patient- and clinician-reported psychological impacts of hypoparathyroidism

Psychological *n* (%)	Comorbidity status		Patient total (*n* = 42)	Clinician total (*n* = 5)
	Has hypothyroidism (*n* = 31)	Does not have hypothyroidism (*n* = 11)		
Anxious/anxiety	26 (84)	8 (73)	34 (81)	5 (100)
Frustration	22 (71)	5 (45)	27 (64)	5 (100)
Depressed/sad	22 (71)	4 (36)	26 (62)	4 (80)
Isolated	15 (48)	3 (27)	18 (43)	1 (20)
Reduced self-confidence/self-image	15 (48)	4 (36)	19 (45)	4 (80)
Reduced self-confidence/self-esteem	11 (35)	1 (9)	12 (29)	4 (80)
Self-image/identity	8 (26)	4 (36)	12 (29)	0 (0)
Irritable/short-tempered	14 (45)	4 (36)	18 (43)	1 (20)
Worry	15 (48)	2 (18)	17 (40)	2 (40)
Anger	11 (35)	3 (27)	14 (33)	2 (40)
Has learned to live with condition	7 (23)	1 (9)	8 (19)	0 (0)
Embarrassed	7 (23)	0 (0)	7 (17)	0 (0)
Fearful about the future	6 (19)	1 (9)	7 (17)	0 (0)
Can't trust/rely on one's body	3 (10)	3 (27)	6 (14)	2 (40)
Feel sorry for oneself	5 (16)	1 (9)	6 (14)	0 (0)
Feel like burden to others	3 (10)	2 (18)	5 (12)	0 (0)
Impatient	2 (6)	3 (27)	5 (12)	0 (0)
Panic attacks	3 (10)	2 (18)	5 (12)	0 (0)
Personality change	5 (16)	0 (0)	5 (12)	0 (0)
Stress	3 (10)	2 (18)	5 (12)	0 (0)
Emotional wreck	3 (10)	0 (0)	3 (7)	0 (0)
Grief/sense of loss	2 (6)	1 (9)	3 (7)	0 (0)
Moody	2 (6)	1 (9)	3 (7)	0 (0)
Argumentative	1 (3)	1 (9)	2 (5)	0 (0)
Feel defeated	1 (3)	1 (9)	2 (5)	0 (0)
Overwhelmed	2 (6)	0 (0)	2 (5)	0 (0)
Feel helpless/hopeless	4 (13)	0 (0)	2 (5)	0 (0)
Mood swings/emotional lability	1 (3)	1 (9)	2 (5)	0 (0)
More emotional	2 (6)	0 (0)	2 (5)	0 (0)
Self-conscious	2 (6)	0 (0)	2 (5)	0 (0)
Upset	1 (3)	1 (9)	2 (5)	0 (0)
Can't trust/rely on one's mind	0 (0)	1 (9)	1 (2)	0 (0)
Staying emotionally composed in public	0 (0)	1 (9)	1 (2)	0 (0)
Emotionally deadpanned/flat	0 (0)	1 (9)	1 (2)	0 (0)
Emotionally drained	1 (3)	0 (0)	1 (2)	0 (0)
Terror/fight or flight response	0 (0)	1 (9)	1 (2)	0 (0)
Humiliation	1 (3)	0 (0)	1 (2)	0 (0)
Lack of motivation	1 (3)	0 (0)	1 (2)	0 (0)

Participants and clinicians reported a total of 9 HP impacts on social life and relationships (Table 4), including reduced ability to participate in social activities (*n* = 33, 79%), a negative effect on relationships (*n* = 32, 76%), and limitations in the types of activities one can participate in (*n* = 24, 57%).

**Table 4:** Patient- and clinician-reported impacts of hypoparathyroidism on social life and relationships

Social life and relationships *n* (%)	Comorbidity status		Patient total (*n* = 42)	Clinician total (*n* = 5)
	Has hypothyroidism (*n* = 31)	Does not have hypothyroidism (*n* = 11)		
Impaired ability to participate in social activities	24 (77)	9 (82)	33 (79)	2 (40)
Relationships	24 (77)	8 (73)	32 (76)	4 (80)
Relationships with family	14 (45)	4 (36)	18 (43)	3 (60)
Relationships with friends	14 (45)	2 (18)	16 (38)	2 (40)
Relationships with spouse/partner	4 (13)	5 (45)	9 (21)	2 (40)
Limited in types of social activities one can participate in	16 (52)	8 (73)	24 (57)	1 (20)
Social withdrawal	17 (55)	5 (45)	22 (52)	0 (0)
Symptoms disrupt social activities	6 (19)	3 (27)	9 (21)	0 (0)
Unable to play same role in family/friendship group	6 (19)	0 (0)	6 (14)	0 (0)
Unable to help out as much in household	4 (13)	0 (0)	4 (10)	0 (0)
Unable to keep up with others	2 (6)	1 (9)	3 (7)	1 (20)
Unable to contribute as much to household income	2 (6)	0 (0)	2 (5)	0 (0)

A higher proportion of participants with hypothyroidism as well as HP reported being less physically active (18/31, 58%) than they used to be/want to be compared with those without this comorbidity (4/11, 36%). Participants with hypothyroidism and HP also reported interference with work productivity (17/31, 55%), and impaired ability to do leisure activities/hobbies (12/31, 39%), compared to those without this comorbidity (1/11, 9%, and 2/11, 18%, respectively). Participants with hypothyroidism and HP reported experiencing the following psychological impacts compared to those without this comorbidity: frustration (22/31, 71%, vs. 5/11, 45%), depressed/sad (22/31, 71%, vs. 4/11, 36%), isolated (15/31, 48%, vs. 3/11, 27%), and worry (15/31, 48%, vs. 2/11, 18%). Participants with hypothyroidism and HP also reported a negative impact on relationships with friends (14/31, 45%) compared with those without this comorbidity (*n* = 2/11, 18%). A lower proportion of participants with hypothyroidism and HP, however, reported being limited in the types of social activities they could participant in (16/31, 52%) and a negative impact on their relationship with their spouse/partner (4/31, 13%) compared to those with HP alone (8/11, 73% and 5/11, 45%, respectively).

#### Preliminary theoretical model

**Fig. 1:**
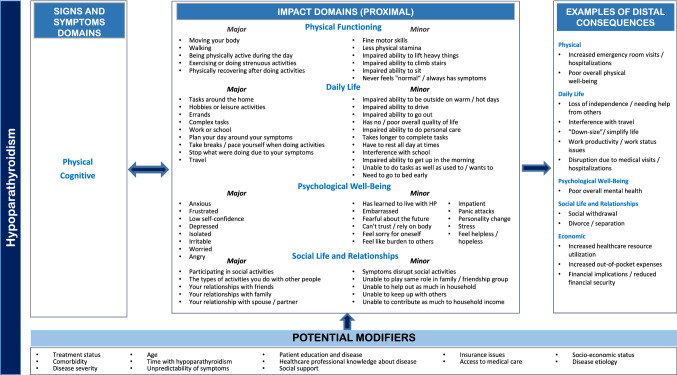
Preliminary theoretical model of the impacts of hypoparathyroidism on quality of life

Based on CE results, a preliminary theoretical model of the signs and symptoms of HP and their impact on QOL was developed (Fig. 1). The model illustrates the hypothesized relationships among the major and minor proximal impacts and distal impacts along with potential mediators/modifiers. Potential modifiers/mediators which may mitigate or amplify the impacts of HP include treatment status, comorbidities, disease severity, age, time with HP, social support, and disease etiology. The preliminary model may require modification based on future psychometric evaluation studies.

### Item generation

Based on the criteria outlined above, 27 impacts were categorized as major and considered for inclusion in the measure. One item meeting the criteria, interference with travel (64%), was excluded from the measure because most people would not have traveled during the past two weeks, the recall period of the measure.

Items were worded as closely as possible to language used by participants to describe their impacts. For each item generated, the findings from the original CE data were reviewed to confirm that the item reflected a concept considered to be important and bothersome by a majority of the participants who reported experiencing it.

### Cognitive debriefing

A total of 16 adults participated in CD interviews. As with the CE sample, the majority (*n* = 12, 75%) were female, and had post-surgical HP (*n* = 11, 69%). The average age was 47 (range 20–63) years. On average, participants had HP for 12 (range 2–50) years, had an average of 3 (median 2, range 0–9) comorbidities, and used 4 (range 1–8) prescription medications.

After the first block of interviews, findings were reviewed, and a decision was made on whether any changes to the HPES-Impact were necessary. This process continued in blocks of four participants until a determination was made that the readability, clarity and relevance of all instructions and items were acceptable based on consensus agreements among respondents in an entire block. A total of four blocks were necessary to refine the HPES-Impact items.

The CD resulted in the validation-ready HPES-Impact, made up of 26 items. Of the 27 impacts categorized as major during the item generation phase, all were retained except for “low self-image”, which CD respondents considered to be synonymous with “low self-confidence.” Items are grouped into stems that ask participants about the impacts of HP for each domain. Response options are based on a 5-point Likert-type scale (response options: “Not at all,” “A little,” “Moderately,” “A lot,” and “Extremely”), which was chosen to ensure that meaningful distinctions could be made among responses for analysis while minimizing the cognitive burden for respondents [31]. The two-week recall allows for the daily variability of symptoms which affect impacts and, based on the data from the CE and CD interviews, is common for this condition. CD participants confirmed the appropriateness and ability to answer for a recall period of this duration.

Table 5 presents representative quotes for each of the 26 HPES-Impact items.

**Table 5:** Exemplary quotes for the items in the HPES-Impact

Items	Exemplary quotes
Physical functioning	
Moving your body (such as arms, legs, neck)	The tetany can make it difficult to move around, because everything is kind of cramped up through the whole cardiovascular system. (205; male, age 35, idiopathic, taking PTH replacement therapy) I sometimes can't drive because my forearms and my hands are too numb to control the steering wheel. Things like doing my hair, having my arms elevated for too long start to really hurt and go numb as well…Even like squatting down to pick up my kids’ toys, my legs go numb. (215; female, age 31, post-surgical)
Walking	It's very difficult and painful, and dangerous, actually, to walk when my legs are numb and tingly. (225; female, age 49, post-surgical, has hypothyroidism, taking PTH replacement therapy) If I walk for long periods of time, especially the next day, I am done with. I get leg pains and I don’t know if they’re necessarily cramps per se, but I get the joint pain and muscle aches, and just, recovery time is a lot longer than it ever would have been before hypopara. (229; female, age 46, post-surgical, has hypothyroidism)
Being physically active during the day	I just can't do as much as I used to do… I don't have enough energy to go on a walk… I'm fairly young, and I—like—I'm like I wasn't expecting that I would have something like this that would really change my energy level and what I can do physically every day. (240; female, age 44, post-surgical, has hypothyroidism) Growing up, all my life, I’ve been into sports, I’ve always enjoyed soccer and other sports, and playing with my children outside. I can’t do it anymore, because of the tingling sensation and the cramping, and the overall just not feeling well, tired, and on top of all that I have a lot of cardiac issues now because of the hypopara that I did not before. (242; female, age 38, post-surgical, has hypothyroidism, taking PTH replacement therapy)
Physically recovering after activities (such as daily activities, social activities)	If I have to go out to go grocery shopping or something, it may take me two days just to recover from going shopping, because it takes so much out of me (243; female, age 60, post-surgical, has hypothyroidism, taking PTH replacement therapy) If I know I need to do something, somehow I could always rise to the occasion, but then I know I’m going to need time to fall apart. If I have something big I have to do, I’m able to know, okay, so on Tuesday I have to do this and this, well, I better hang low on Sunday and Monday and know for sure that nothing could be scheduled, and depending… [on] how intense it is what I’m doing, how long it’s going to take me to get back to what I—where I should be. (214; female, age 57, post-surgical)
Daily life	
Tasks around the home (such as cooking, cleaning, outdoor work)	It used to be no big deal to vacuum my whole house. Now I vacuum just a room. Yes, I just get worn out quicker and my muscles start hurting and they’re sore. (235; female, age 56, post-surgical, has hypothyroidism) I stopped cooking altogether. I love to cook and I stopped cooking altogether because the recipes are just too hard to figure out, and I just couldn’t get the energy and the interest, and I started eating a lot of frozen food. (216; female, age 61, post-surgical, has hypothyroidism, taking PTH replacement therapy)
Hobbies or leisure activities	I used to do a lot more creative things. I used to paint. Now I just don't have the energy for it. So, I might do some small little project and then I won't do anything again for maybe a month or two and then I have to force myself to get into it. (213; female, age 71, post-surgical, has hypothyroidism) My one thing I just love to do is I’m in choir…But on Wednesday nights when it’s getting time to go, because the practice isn’t until 7:30 in the evening, I am so tired and so usually hurting so much that it’s like it’s such an effort to go. (236; female, age 61, post-surgical, has hypothyroidism, taking PTH replacement therapy)
Errands (such as shopping)	[Due to tingling sensations] I don’t really do much. I’m basically—it’s difficult to get up and do anything, to get up out of bed and do grocery shopping or do anything. (239; female, age 58, post-surgical, has hypothyroidism, taking PTH replacement therapy) Well, the worst feeling one is the cramping, and being sick. I would say not feeling good; fatigue is what you called it, but yeah, and then lack of sleep…Because it’s interrupting my life, I mean day-to-day functions. You just, whether you’re going grocery shopping or you’ve got appointments…and you can’t, I mean it just … You don’t feel good. (241; female, age 52, post-surgical, has hypothyroidism)
Complex tasks (such as paying bills, keeping track of appointments)	My calcium levels have been more stable, so—but again, if I have something like stomach flu and I have problems with [calcium] absorption, then I’ll find myself kind of like, okay, this is not the day to balance your checkbook. (217; female, age 62, post-surgical, has hypothyroidism, taking PTH replacement therapy) I’m unable to—because of the brain fog, put things together, like multi-task, timing with food. (218; female, age 61, post-surgical, has hypothyroidism)
Work or school	I just get really tired, really fatigued. It's not even tired, it's beyond tired. It's like you never really feel like you get your strength back. (213; female, age 71, post-surgical, has hypothyroidism) I am very tired. Yes, like I hit a wall and I just—I don't know how I can go any further… just so fatigued all day long. (225; female, age 49, post-surgical, has hypothyroidism, taking PTH replacement therapy)
Plan your day around your symptoms	The most challenging impact is how am I going to get through the days and I guess coping with the whole—getting up every day and realizing that I have this chronic condition and how am I going to deal with it, how am I going to plan the day. (209; female, age 76, post-surgical) I'm getting to a point in the day where it's starting to get to be weighing on me a little bit. I still have some things I want to try to do this afternoon, so I'm already wondering whether that's a good idea or not…I try to do what I can in the morning and then not plan a whole lot in the afternoon and evenings. (202; male, age 59, post-surgical, has hypothyroidism)
Take breaks or pace yourself when doing activities	I can't just clean the house in one day like I used to. I have to take breaks in between. (203; female, age 29, post-surgical, has hypothyroidism) I have to pace myself, I have to be very aware of how much energy I'm expending and what the temperature is outside and make sure I have my extra calcium and I bring coconut water with me in a cooler and things like that, make sure I have electrolytes with me all the time. (211; female, age 39, post-surgical, has hypothyroidism, taking PTH replacement therapy)
Stop what you were doing due to your symptoms	If I was to do dishes or something like that, I'd have to stop and wait for it [the tingling] to quit before I could continue. Yes. It could affect whatever, if I'm doing something with my hands. Maybe even driving, I noticed a couple of times it was really bad. I had to stop over, yes. (202; male, age 59, post-surgical, has hypothyroidism) It [muscle cramping] takes you out of the moment, obviously. If I'm just doing something and I get one, it's just like all my focus goes to getting rid of it, and it's usually in my legs or my—or like my calves or my feet is where I get them the most. (220; female, age 40, post-surgical, has hypothyroidism)
Psychological	
Anxious	I have anxiety that I didn't used to have…with the first year and a half, when my calcium would drop and I would get those tetany episodes, I think I almost developed post-traumatic stress because every time I would start tingling, I would kind of go into a panic and think, “Oh no, not again. You know, not again. I'm going to have to go to the emergency room.” (233; female, age 58, post-surgical, has hypothyroidism) Mentally, I just don't think clear anymore, like I just don't feel smart. Then that just has a great onset of anxiety and depression. It just wears you down. (201; male, age 26, idiopathic, has hypothyroidism, taking PTH replacement therapy)
Frustrated	I get angry about not being able to do things that are—having felt like this for 41 years, it’s very frustrating. (239; female, age 58, post-surgical, has hypothyroidism, taking PTH replacement therapy) Part of the reason it's so frustrating is that there's no way to gauge when your calcium's going to drop. You can anticipate that maybe I'm a little bit more active than I normally am so maybe I ought to take another calcium pill, but you can't always figure it out and sometimes it hits you out of the blue and you just get so, so fatigued. (238; female, age 69, post-surgical, taking PTH replacement therapy)
Low self-confidence	Sometimes having the brain fog business makes me self-conscious about being around people too, because if I can’t remember something, it makes me feel real stupid. I’ve always been a kind of a person who lives in their brain and really appreciates intellectual endeavors and stuff, and I feel a lot stupider than I used to, and that’s kind of hard. (237; female, age 69, post-surgical, has hypothyroidism, taking PTH replacement therapy) I was always someone who really counted on and relied on my mind, and when it started going wonky on me, I found it very disconcerting and stressful and a real challenge to my self-identity. (206; male, age 28, idiopathic, taking PTH replacement therapy)
Depressed	It’s depressing that I can’t do things like stay up late and go to big events or parties or even family functions. My whole family has to do things early because I have to go to bed early, because I didn’t sleep the night before. It makes me depressed and sad. (230; female, age 45, post-surgical, has hypothyroidism) I've been very angry and very depressed over periods of time, not just a day here and a day there, but it'll go on for weeks at times, especially if I'm not feeling well, and I don't seem to—this is particularly before PTH replacement therapy, and I would go on for days and days and days and days of not feeling well, and it's like nothing seemed to make any difference to help me feel better. (223; female, age 66, taking PTH replacement therapy)
Isolated	That was isolation that I was talking about… because I don't get to go out liked I used to, when I do go out, I don't have a lot to talk about and I don't—I've never met anyone else who has hypopara and nobody else knows anyone with it so they don't understand. (211; female, age 39, post-surgical, has hypothyroidism, taking PTH replacement therapy) The isolation of it all. There’s nobody knowing what you’re talking about when…You talk about it, and people kind of—sometimes even take it as a joke because they’re like, “Well, it can’t be that serious if nobody else has it,” you know? People will just be like, just laugh about it. I tell them about it and they’ll see me and I look—if you look at me firsthand, you don’t think anything’s wrong with me. (222; male, age 28, post-surgical, has hypothyroidism)
Irritable	My major signs and symptoms actually come first with being very kind of irrational and irritable… [before hypoparathyroidism] I would never snap at my kids verbally for something simple, where now it’s—I can yell at them for doing something that they don’t deserve to be yelled at for. (229; female, age 46, post-surgical, has hypothyroidism) It makes me crabby. Less patient than I might be. And cranky. (207; female, age 75, post-surgical, taking PTH replacement therapy)
Worried	I worry if my calcium level starts to fall…I start feeling quite (inaudible) at first and then other symptoms follow. (205; male, age 35, idiopathic, taking PTH replacement therapy) I'm only 30 years old. I feel like if this is how I feel now, I worry, is it going to be worse when I get older? Is it going to progress? I just think, if this is hard to feel now, how I'm going to feel 40 years from now? That is my biggest worry. (203; female, age 29, post-surgical, has hypothyroidism)
Social life and relationships	
Participating in social activities	Is it worth it, do I want to go to this event, how am I going to feel after, what else do I need to accomplish today? Sort of that picking and choosing of where to spend your time and energy, and—yes, and sort of what did I do before or what do I have coming up that I’d like to maybe preserve my energy, if that makes sense. (221; female, age 33, has hypothyroidism, taking PTH replacement therapy) What I say is, “You can pencil me in. Don’t ever pen me in,” because I don’t know. I want to be normal. I want to say I’m going to be there, but I can tell you that my body will tell me that day if I can physically make it or not. (231; female, age 49, post-surgical, has hypothyroidism, taking PTH replacement therapy)
The types of activities you do with other people	I can’t do things like stay up late and go to big events or parties or even family functions. My whole family has to do things early because I have to go to bed early. (230; female, age 45, post-surgical, has hypothyroidism) If there's anything that involves being outside…if there's stuff that involves picnics or whatever outside, there'll be things that definitely we'll pass on because I can't handle it. (238; female, age 69, taking PTH replacement therapy)
Relationships with friends	When I don't feel well and I end up canceling plans or not going out because I know that it's going to be too hard for me, people kind of just stop wanting to do things or asking me to do things. (225; female, age 49, post-surgical, has hypothyroidism, taking PTH replacement therapy) When you are not able to go out and participate in all the activities that you used to be able to do, then people forget about you…I mean, they may not totally forget about you. Like they might think, "Oh I wonder how (name)’s doing," but it's not like these people are in my life anymore. (213; female, age 71, has hypothyroidism, post-surgical)
Relationships with family	I went on a cruise with my family and my children said something about, “I wish you could walk further, walk more.” It’s just—they don’t understand how painful it is. (239; female, age 58, post-surgical, has hypothyroidism, taking PTH replacement therapy) I rely on them a lot more than I used to which is causing them strain, and I have a lot less patience and a lot less positive ability to kind of be the leader of the family. Kind of a funny way to put it, but I’m usually the one who brings like the energy and the direction and the enthusiasm…I’m just simply unable to do that anymore. (218; female, age 61, post-surgical, has hypothyroidism)
Relationship with spouse/partner	Being a caretaker has been really hard for my wife. (206; male, age 28, idiopathic, taking PTH replacement therapy) My husband, he is completely understanding about all this. He's coming to terms with life—the new type of life we have, but even sometimes he gets frustrated because—yesterday I felt so well and I was able to go ride the bikes with the kids and things like that, and today I can't get out of bed. (215; female, age 31, post-surgical)

## Discussion

The data from this research indicates that the impact burden among patients with HP is substantial, even when on conventional and PTH replacement treatment. This burden is evident in the wide range of impacts as well as their reported frequency, severity, and bothersomeness.

Notably, over 40% (*n* = 18, 43%) of participant-reported interference with work productivity that they attributed to the physical and/or cognitive symptoms experienced with HP. This represented the vast majority of participants who were currently employed (*n* = 21, 86%). An additional one-third of the sample (*n* = 14, 33%) reported no longer being able to work due to their HP symptoms. These findings underscore the debilitating nature of this condition and suggest that HP may have economic implications which should be quantitatively examined in future research.

Higher proportions of participants with hypothyroidism reported experiencing several daily life and psychological impacts compared with participants without this comorbidity. This may be due to an increased burden resulting from experiencing these chronic conditions simultaneously. It should be further noted that not all impacts were disproportionately experienced by those with both HP and hypothyroidism. There may be several explanations for this, including the influence of other comorbidities on patients without hypothyroidism, the severity of HP among some of those without hypothyroidism, the source of one’s HP, length of time with HP, and/or differences in treatment regimens among patients for both their HP and hypothyroidism. Regarding this latter point, since the focus of the study was on HP, data was not collected on the specific treatments participants were taking to manage their hypothyroidism. Further research with a larger study sample and a quantitative study design would be appropriate to further investigate the potential differences in QOL and their causal factors between these subgroups.

It is anticipated that a brief, disease-specific validated measure of the impacts of HP on patients may benefit clinical care by concretely assessing their specific effects on QOL, and by providing standardized language to describe the nature and severity of these impacts in individuals. This is especially important given the wide range and severity of both the symptoms and corresponding impacts experienced among patients with HP. The HPES-Impact measure provides a convenient way for clinicians to determine where their individual patients fall within this range, facilitating provider-patient discussions and treatment decisions, with a tool that captures the patient perspective. The measure may also be used in future research to evaluate disease burden or the impacts of treatment efficacy on HRQOL.

This study importantly provides evidence for content validity of the impacts captured in the HPES-Impact. Previous disease-specific measures that have been developed for this condition have focused primarily on symptoms and not captured the broad spectrum of both symptom and disease impacts [32]. We believe the HPES-Impact measure is the first disease-specific measure to capture the broad spectrum of disease impacts experienced by adult patients with HP.

As with all research, there are study limitations to consider when interpreting findings. These have been noted in a prior publication but should be reiterated here [28]. While efforts were made to recruit a diverse sample of participants, the majority were female and had post-surgical HP, which is similar to the sample compositions in other studies of patients with this condition [5, 8, 33]. The majority of participants also had hypothyroidism, which, as previously noted, is a common comorbidity with this condition [6]. Although none of the participants in the CE sample had HP with an autoimmune etiology, two participants with autoimmune HP were included in the CD sample, and both confirmed that the measure items were relevant and that the recall period was appropriate based on their experience with the condition. In addition, most participants were white/Caucasian, and it was a US-based population. However, the clinical experts from Denmark reported observing similar impacts among their patients, and studies describing patients with HP in Canada, India and multiple European countries provide evidence that the measure may be appropriate for assessments in other populations [5, 7, 18, 34, 35]. Cultural and linguistic validation of the measure would be required for use in non-US populations. This study was also limited to an adult population. Future research should examine the impacts of HP for children, as impacts may differ. Given the impacts reported by adults on work, for example, it is important to assess if school attendance and work are affected as well.

Given the sample sizes within each CE subgroup and the differences in the numbers of participants within the hypothyroidism vs. non-hypothyroidism subgroups (*n* = 31 vs. *n* = 11), study findings based on subgroups should be interpreted with caution. There were also variations within each subgroup that may impact the burden of illness, such as other comorbidities, duration of time with HP, and a high proportion of participants without hypothyroidism who were taking PTH replacement therapy (8/11, 73%). A quantitative survey study would be appropriate to further explore variations among subgroups based on treatment status, comorbidities, and other potential modifiers such as gender, age, disease etiology, and length of time with the condition.

Lastly, while several study participants reported that they had experienced a reduction in the frequency and severity of impacts due to their treatment, it was not possible to systematically assess these treatment benefits due to the potential for recall bias. It is anticipated that the HPES-Impact will serve as a useful tool for measuring responsiveness to treatment in patients who can be assessed before and after initially starting treatment or changing treatment regimens pending research to evaluate its measurement properties.

## Conclusions

A better understanding of the impact burden experienced by patients with HP is important to inform clinician treatment decisions and address unmet treatment needs. This study provides evidence of content validity for the validation-ready HPES-Impact, which includes 26 items to be completed by adults ages 18 years and older with HP, across four domains of QOL: Physical Functioning, Daily Life, Psychological Well-Being, and Social Life and Relationships. Additional research is needed to assess the measurement properties of the HPES-Impact in patients with HP to determine whether it is a reliable measure. To the best of our knowledge, the HPES-Impact measure is the first disease-specific measure to capture the broad spectrum of disease impacts experienced by adult patients with HP.

## Data Availability

The data for the research presented in the publication may be available from the corresponding author on reasonable request.
